# Phenotypic and Genotypic Characteristics of Adult‐Onset Glutaric Aciduria Type 1: Report of Two Cases and a Literature Review

**DOI:** 10.1002/brb3.70281

**Published:** 2025-02-18

**Authors:** Luhua Wei, Jieyu Li, Zhiying Xie, Ying Zhu, Jing Chen, Yawen Zhao, Yun Yuan, Yining Huang, Yanling Yang, Zhaoxia Wang, Jing Chen

**Affiliations:** ^1^ Department of Neurology Peking University First Hospital Beijing China; ^2^ Department of Radiology Peking University First Hospital Beijing China; ^3^ Department of Neurology Peking University Sixth Hospital Beijing China; ^4^ Beijing Key Laboratory of Neurovascular Disease Discovery Beijing China; ^5^ Department of Pediatrics Peking University First Hospital Beijing China

**Keywords:** adult‐onset, glutaric aciduria type 1, hyperhomocysteinemia, subependymal lesions

## Abstract

**Introduction:**

Glutaric aciduria Type 1 (GA‐1) is an autosomal recessive inherited disorder caused by *GCDH* variations. GA‐1 is a rare disease that typically manifests in infancy and early childhood, with adult‐onset cases being even rarer. Currently, data on the clinical and genetic characteristics of adult‐onset GA‐1 remain limited.

**Methods:**

We hereby reported two new cases of adult‐onset GA‐1 and systematically summarized reported studies to investigate its genotypic and phenotypic features.

**Results:**

Patient 1 presented with seizures as the onset symptom. Patient 2 exhibited recurrent stroke‐like episodes. Brain magnetic resonance imaging showed subependymal lesions. Urine organic acid analyses were performed since both patients had hyperhomocysteinemia (HHcy) and found significantly elevated glutaric acid and 3‐hydroxyglutaric acid. Genetic analysis further identified biallelic missense variants in *GCDH* in both patients (Patient 1: c.383G> A, c.937C> T; Patient 2: c.533G> A, c.1205G> A). A literature review found seven cases and 12 variants in adult‐onset GA‐1. Most of them showed nonspecific neurological manifestations. The most common symptoms were cognitive impairment and headache. Subependymal lesions have been reported in five of seven cases. One of them also had HHcy. All adult‐onset GA‐1 cases were high excretors. All *GCDH* variants are located in nonactive binding regions.

**Conclusion:**

This study characterized the phenotype of adult‐onset GA‐1 emphasizing subependymal lesions and the coexistence of HHcy. The latter might suggest the influence of environmental factors on the age of onset. No clear genotype–phenotype correlation was found.

## Graphical abstract

This study reported two new cases and characterized the phenotype and genotype of adult‐onset glutaric aciduria Type 1 by literature review, emphasizing subependymal lesions and the coexistence of hyperhomocysteinemia. No clear genotype–phenotype correlation was found in adult‐onset glutaric aciduria Type 1.

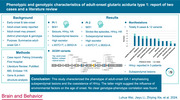

## 1 Introduction

Glutaric aciduria (acidemia) Type 1 (GA‐1, OMIM:231670) is an autosomal recessive inherited disease caused by variations in the *GCDH* gene, resulting in a deficiency of glutaryl‐CoA dehydrogenase. This enzyme is crucial for the metabolism of lysine, hydroxylysine, and tryptophan. Deficiency in this enzyme leads to the accumulation of metabolic products such as glutaric acid (GA), glutarylcarnitine (C5DC), and 3‐hydroxyglutaric acid (3‐OH‐GA) (Boy et al. 2023). GA‐1 is rare, with an estimated prevalence of 1/90,000 to 1/120,000 (Boy et al. 2023). Depending on the urinary GA concentration, two biochemical subgroups have been arbitrarily defined as low excretor (LE) and high excretor (HE) (Boy et al. 2023). Most cases occur in the early childhood. Affected infants are mostly asymptomatic or only develop nonspecific neurologic symptoms at disease onset. Without treatment, most patients will suffer an acute encephalopathic crisis triggered by fever, vaccination, surgery, or other stressors between age 3 and 36 months (Boy et al. 2023; Han et al. 2021).

Onset after 6 years is referred to as late‐onset GA‐1, which differs from the classical form and is mainly characterized by nonspecific symptoms such as headache, dizziness, transient ataxia, impaired fine motor skills, or fainting following exercise (Boy, Heringer, Brackmann, et al. 2017). Late‐onset GA‐1 accounts for about 10%–20% of cases (Boy, Heringer, Brackmann, et al. 2017), among which adult‐onset (≥ 18 years) cases are even rarer. To date, only seven adult‐onset GA‐1 cases have been reported in the literature (Bähr et al. 2002; Boy, Heringer, Brackmann, et al. 2017; Gelener et al. 2020; Herskovitz et al. 2013; Külkens et al. 2005; Pierson et al. 2015; Sonmez et al. 2007). Most cases were insidious and none of them developed acute encephalopathic crisis. Adult‐onset patients may present with different phenotypes from children such as stroke‐like episodes and subependymal lesions. Although it remains questionable whether adult‐onset GA‐1 forms a disease variant, its clinical implication is obvious because patients might present to an adult neurologist without a past childhood history.

To better characterize adult‐onset GA‐1, we report two cases here and summarize the clinical phenotype, biochemical phenotype, and genotype from published literature.

## 2 Materials and Methods

### 2.1 Case Report

This study has been approved by the health authority ethical committee of Peking University First Hospital. Two unrelated patients with adult‐onset GA1 diagnosed in Peking University First Hospital were enrolled. The patients were interviewed and examined by at least two experienced neurologists. All clinical records and results of laboratory examination and neuroimaging were collected. Genomic DNA extracted from peripheral blood samples was used for downstream procedures. Whole exome sequencing was conducted with support from Running Gene Inc. (Beijing, China). The DNA sequencing library construction utilized the MGIEasy DNA Library Prep Kit following the manufacturer's protocols to produce DNA nanoballs. Burrows–Wheeler Alignment Maximal Exact Matches was used for read mapping onto the hg38 human genome as a reference. Variants with a quality score below 20 were excluded, and 95% of the targeted bases were covered sufficiently to meet our quality thresholds for identifying single nucleotide polymorphisms and small insertions or deletions. Variant calling for single‐nucleotide variants and insertions or deletions was performed using GATK (https://software.broadinstitute.org/gatk/), while variant annotation was accomplished using ANNOVAR (http://www.openbioinformatics.org/annovar/annovar_region.html). All potential disease‐associated variants were meticulously reviewed in the Integrative Genomics Viewer (https://software.broadinstitute.org/software/igv/). Sanger sequencing confirmed the heterozygous status of the variants in the probands and their parents, respectively.

### 2.2 Literature Review

#### 2.2.1 Search Strategy

A review of the literature was then performed on PubMed and Embase up to May 2024 with the following search terms in all fields: “glutaric acidemia type 1,” “glutaric aciduria type 1,” “glutaric acidemia type I,” or “glutaric aciduria type I,” or “glutaryl‐CoA dehydrogenase deficiency.” For patients with subependymal lesions, “subependymal” was added in the query box. Abstracts, conference papers, and preprints were excluded. Publication language was restricted to English. Totally 880 articles were found on PubMed and 116 exclusively on Embase.

#### 2.2.2 Inclusion and Exclusion Criteria

All cases with a clear diagnosis of GA‐1 according to the guidelines (Boy, Mühlhausen, Maier, et al. 2017; Boy et al. 2023) were reviewed. Those who developed the first symptom other than macrocephaly after the age of 18 years were included. Symptoms were defined as movement disorders, cognitive impairment, focal neurological symptoms, or any nonspecific symptoms without other identified causes. The cases with no clear disease onset age were excluded. The cases with no description of subependymal lesions were not included in the imaging analysis.

#### 2.2.3 Data Extraction

All data extracted were documented in an Excel spreadsheet. Data were collected on demographic information, clinical presentation, diet, macrocephaly, homocysteine (HCY), electrophysiological studies, brain magnetic resonance imaging (MRI), plasma C5DC and free carnitine, urine GA and 3‐OH‐GA, and genotype.

LE phenotype was defined as GA excretion of < 100 mmol GA/mol creatinine and HE phenotype as GA excretion of > 100 mmol GA/mol creatinine (Baric et al. 1999). Brain MRIs were reviewed by two experienced neurologists if images were given. The number of subependymal lesions was defined as “multiple” if the number of nodules > 2 and could not be counted given limited data. The diameter was documented from the text or measured from the images if a scale bar was given.

### 2.3 Prediction of the Phenotype Using Protein Structure

To correlate the adult‐onset phenotype of the pathogenic variant to the GCDH protein structure, we annotated all reported variants for adult‐onset GA‐1 on the 2D and 3D diagrams of GCDH protein (PDB ID: 1SIR). 3D structure presentation was generated with the PyMOL Molecular Graphics System, Version 2.5.0 Schrödinger, LLC.

## 3 Results

### 3.1 Case Report

#### 3.1.1 Patient 1

The patient was a 41‐year‐old Chinese man who experienced an afebrile seizure 12 days before admission. He suddenly lost consciousness with bilateral arms twitching for 2 min. No turning of the head or eyes was noticed. The patient received three vaccines within 2 months, the last one vaccinated 2 days before the seizure. His past medical history was unremarkable and he did not have macrocephaly. School performance was average during childhood. He was not a vegetarian although he preferred a diet with fruits and vegetables. The family history was negative. On physical examination, the overall appearance and stature were normal. The neurological examination revealed a normal mental status and motor function. A slight horizontal nystagmus was detected. Deep tendon reflexes appeared brisk without pathological signs (Table 1).

**TABLE 1:** Clinical phenotype, biochemical phenotype, and genotype of adult‐onset GA‐1.

Reference	Sex	Age at onset (years)	Age at diagnosis (years)	Clinical presentation before treatment (in chronological order)	Clinical presentation after treatment	Macrocephaly	HCY	Electrophysiological studies	Diet before treatment	Plasma C5DC (µmol/L)	Plasma free carnitine (µmol/L)	Urine GA at diagnosis (mmol/mol Crea)	Urine 3‐OH‐GA at diagnosis (mmol/mol Crea)	Enzyme activity	Genotype
Patient 1	Male	41	41	Seizure, nystagmus, hyperreflexia	No recurrent seizure	No	High	EEG: occipital asymmetry with lower voltage on the left, no epileptiform discharges. NCS: diffuse mild slowing of sensory and motor nerve velocity	Few meats or fruit	0.78	3.87	3711 (fold change vs. control)	74.57 (fold change vs. control)	ND	c.937C> T, p.(Arg313Trp); c.383G> A, p.(Arg128Gln)
Patient 2	Male	51	51	Repetitive stroke‐like episodes	Cognitive impairment, glomerular injury	Yes	High	EEG: background slowing, continuous slowing, regional sharp waves	No restriction	0.16 (51y) → 0.67 (64y)	2.77 (51y) → 15.06 (64y)	2186 (51y) → 2206 (64y) (fold change vs. control)	11.89 (51y) → 9.99 (64y) (fold change vs. control)	ND	c.533G> A, p.(Gly178Glu); c.1205G> A, p.(Arg402Gln)
Gelener et al. (2020)	Female	35	35	Headache, memory decline, mild anxiety & depression	ND	ND	ND	ND	Mediterranean diet with meat consumption at least once a week.	1.10	1.99	7176	ND	ND	c.1204C> T, p.(Arg402Trp); homozygous
Boy, Heringer, Brackmann, et al. 2017	Male	61	71	Repetitive cerebral ischemia, intention tremor, confusion, progressive dementia, possible non‐convulsive seizures, incontinence	ND	Yes	High	EEG: mild focal abnormalities, no epileptiform discharges. NCS: polyneuropathy	ND	ND	ND	2149	99 (follow‐up)	ND	c.1204C> T, p.(Arg402Trp); c.1262C> T, p.(Ala421Val)
Pierson et al. (2015)	Female	49	55	Episodic lower extremities paresthesia, restless legs, involuntary movements, urinary incontinence, lower extremities spasticity, inattentiveness	ND	ND	ND	NCS: normal	ND	0.28	4	344	160	ND	c.1219C> G, p.(Leu407Val); c.848del, p.(Leu283Argfs*8)
Herskovitz et al. (2013)	Male	26	56	Feet pain, lower extremities weakness, speech disturbance, incontinence, cognitive impairment, lower extremities hyporeflexia, pes cavus	Cognitive decline, no changes in polyneuropathy	ND	ND	NCS: demyelinating and axonal polyneuropathy	ND	0.33	0.63	ND	ND	ND	c.301G> A, p.(Gly101Arg); homozygous
Sonmez et al. (2007)	Male	20	20	Headache, hyperreflexia	Headache improved	ND	ND	EEG: sharp and slow waves in the right frontotemporal region	ND	ND	ND	ND	ND	ND	ND
Külkens et al. (2005) and Boy, Heringer, Brackmann, et al. 2017	Male	35	66	Headaches, tremor, seizures, ataxia, orofacial dyskinesia, hallucinations, aggressive behavior, dementia, hyporeflexia	Gait improved, MMSE 8 to 16	Yes	ND	ND	ND	0.50	6	1595	109	< 1%	c.1147C> T, p.(Arg383Cys); homozygous
Bähr et al. (2002)	Female	19	19	Headaches, nystagmus, upward gaze palsy, convergence paralysis, fine motor dysfunction, hyperreflexia	Headache improved	No	ND	EEG: general paroxysmal dysrhythmia; NCS: normal	ND	0.18	4	1274	134	0%	c.219del, p.(Tyr74Thrfs*68); c.394C> G, p.(Arg132Gly)

Abbreviations: 3‐OH‐GA, 3‐hydroxyglutaric acid; C5DC, glutarylcarnitine; EEG, electroencephalogram; GA, glutaric acid; GA‐1, glutaric aciduria type 1; HCY, homocysteine; MMSE, mini‐mental state examination; NCS, nerve conduction study; ND, not determined.

**FIGURE 1:**
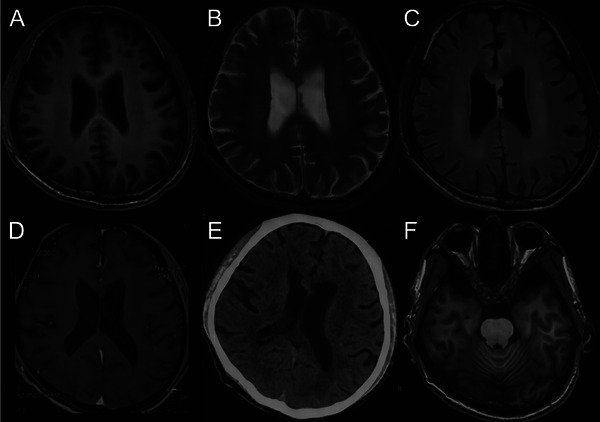
Brain imaging of Patient 1. Multiple subependymal nodules were seen in lateral ventricles and septum pellucidum, predominantly at the roof level (A‐C). The nodules showed no enhancement on gadolinium magnetic resonance imaging (MRI) (D). A computed tomography scan found no calcification (E). Brain MRI also showed periventricular, subcortical, and deep white matter changes with sparing of U‐fibers (C) and temporal poles (not shown). Mild frontotemporal hypoplasia (F), brain atrophy, and ventricular enlargement were noticed.

Routine tests were unremarkable. Fasting blood glucose, lactic acid, transaminase, creatinine, and creatine kinase were all within normal limits. Both blood total HCY (96 µmol/L, normal range 6–17 µmol/L) and ammonia (61 µmol/L, normal range < 60 µmol/L) were elevated, and vitamin B12 (47 pmol/L, normal range 133–675 pmol/L) decreased. The methylenetetrahydrofolate reductase (*MTHFR*) genotype was 677TT. Cerebrospinal fluid (CSF) protein was 0.75 g/L. No interictal epileptiform discharges were observed on the electroencephalogram (EEG). A nerve conduction study demonstrated diffuse mild slowing of sensory and motor nerve velocity. The brain MRI showed multiple nodules on the ventricular wall and the septum pellucidum. No enhancement was observed. There were mild but diffuse white matter lesions in the subcortical and periventricular areas. Mild frontotemporal hypoplasia and brain atrophy were present (Table 2, Figure 1). An inherited metabolic disorder was suspected due to hyperhomocysteinemia (HHcy) and thus urinary organic acids and analysis of blood amino acids and acylcarnitine were conducted, which revealed an increased blood C5DC, decreased blood free carnitine, and a significant elevated urine GA and 3‐OH‐GA (Table 1). Whole exome sequencing found heterozygous variants of c.937C> T, p.(Arg313Trp) (from his mother; his father had passed away) and c.383G> A, p.(Arg128Gln) in the *GCDH* gene. No novel variations were found (Table 1). No other variations consistent with the phenotype were found.

**TABLE 2:** Imaging features of adult‐onset GA‐1 and all GA‐1 patients with subependymal lesions.

		Subependymal lesions (per images given in the reference)							
Reference	Age at imaging (years)	Number	Maximum diameter (mm)	Morphology	Distribution	White matter changes	Gray matter changes	Frontotemporal hypoplasia	Other features
**Adult‐onset GA‐1 (including two patients without subependymal lesions)**									
Patient 1	41	7	10	Nodular, cystic	Lateral ventricles, especially at the roof level; septum pellucidum	Periventricular, subcortical, and deep white matter, Fazekas grade 3, sparing of U‐fibers and temporal poles	No	Mild	Mild brain atrophy, enlargement of lateral and third ventricles
Patient 2	From 51 to 64	7 (64y)	8 (64y)	Nodular	Lateral ventricles, especially at the roof level on the medial border; septum pellucidum	Periventricular, subcortical, and deep white matter, Fazekas grade 3, involving the U‐fibers	Multiple infarctions in the right frontal lobe, occipital lobe, and putamen. Left intracranial vertebral artery thin → occlusion	Moderate	Moderate brain atrophy, enlargement of lateral and third ventricles
Gelener et al. (2020)	35	Multiple	ND	Nodular, cystic	Lateral ventricles, especially at the roof level on the medial border; septum pellucidum	Periventricular, Fazekas grade 2, sparing of U‐fibers	No	Mild	Mild brain atrophy
Boy, Heringer, Brackmann, et al. 2017	From 61 to 73	From 2 to 5	From 10 to 13	Nodular, incipient	The roof and base of anterior horns; septum pellucidum	Periventricular and deep white matter, Fazekas grade 2, sparing of U‐fibers (infarction not included), progressive. Left parietal border zone infarction	Progressive multiple infarctions including the brain stem, right lenticular nucleus and caudate, and left medial temporo‐occipital gyrus. Left MCA stenosis	Severe, and remained stable	Moderate brain atrophy
Pierson et al. (2015)	55	Multiple	ND	Cystic	Bilateral ventricles	Periventricular and deep white matter, Fazekas grade 3, sparing of U‐fibers	No	Moderate	Mild brain atrophy
Herskovitz et al. (2013)	From 56 to 59	Multiple	ND	Cystic	Lateral ventricles, especially at the roof level on the medial border; septum pellucidum	Periventricular and deep white matter, Fazekas grade 3, U‐fibers involved	No	Yes	Moderate brain atrophy, communicating hydrocephalus
Sonmez et al. (2007)	20	No	No	No	No	Periventricular and deep white matter, sparing of U‐fibers	ND	ND	ND
Külkens et al. (2005) and Boy, Heringer, Brackmann, et al. 2017	66	1	17	ND	ND	Periventricular and deep white matter, Fazekas grade 3, U‐fibers involved	No	Moderate	Moderate brain atrophy
Bähr et al. (2002)	19	No	No	No	No	Periventricular and deep white matter, Fazekas grade 3, sparing of U‐fibers	Putamen, tegmentum, superior colliculi	ND	ND

Reference	Sex	Age at onset (years)	Age at imaging (years)	Urine GA at diagnosis (mmol/mol Crea)	Genotype	Subependymal lesions (per images given in the ref.)			
						Number	Maximum diameter (mm)	Morphology	Distribution
**Other late‐onset GA‐1 with subependymal lesions**									
Boy, Heringer, Brackmann, et al. 2017	Female	≤ 18	31	1590 (follow‐up)	c.219del, p.(Tyr74Thrfs*68); c.394C> G, p.(Arg132Gly)	2	3	ND	ND
Boy, Heringer, Brackmann, et al. 2017	Female	≤ 25	37	High excretor	c.1093G> A, p.(Glu365Lys); homozygous detected in 4 other family members	Multiple	19	Nodular, cystic	Bilateral ventricular roofs, septum pellucidum
Harting et al. (2009) and Boy, Heringer, Brackmann, et al. 2017	Female	8.5	From 8 to 15	973	c.383G> A, p.(Arg128Gln); c.1240G> A, p.(Glu414Lys)	From 0 to 7	1.5	Nodular, incipient	Bilateral ventricular roofs
Korman et al. (2007)	Male	≤ 30	30	728	c.877G> A, p.(Ala293Thr); c.578_579insTCA, p.(Thr193_Arg194insHis)	Multiple	ND	Nodular	Bilateral ventricular roofs
Külkens et al. (2005) and Boy, Heringer, Brac et al. 2017	Male	14	14	High excretor	c.262C> T, p.(Arg88Cys); homozygous	Multiple	3	Nodular	Bilateral ventricular roofs
**Early‐onset GA‐1 with subependymal lesions**									
Patel et al. (2018)	Female	ND	From 22 to 29	ND	c.1204C> T, p.(Arg402Trp); homozygous	Multiple	ND	Nodular	Bilateral ventricular roofs
Boy, Heringer, Brackmann, et al. 2017	Male	≤ 3 months	From 14 to 22	2670	c.554G> C, p.(Gly185Ala); c.526T> C, p.(Cys176Arg)	From 1 to 2	From 2 to 3	Nodular	ND
Karimzadeh et al. (2015)	ND	8 patients, ≤ 16 months	≤ 77 months	1 patient normal, 2 patients elevated	c.706T> C, p.(Phe236Leu); c.730G> T, p.(Gly244Cys); c.743C> T, p.(Pro248Leu); c.1286C> T, p.(Thr429Met); all homozygous (only 5 patients had gene results)	ND	ND	ND	ND

*Note*: Variants in the *GCDH* gene were described in relation to coding DNA reference sequence NM_000159.4 and protein reference sequence NP_000150.1.

Abbreviations: GA, glutaric acid; GA‐1, glutaric aciduria type 1; ND, not determined.

The patient was given a low‐lysine diet, l‐carnitine, and levetiracetam. Folic acid, vitamin B6, and mecobalamin were also prescribed for HHcy. Levetiracetam was discontinued after 3 months. A follow‐up after 2 years revealed no recurrent seizures or any other neurological symptoms.

#### 3.1.2 Patient 2

This patient has been previously reported by us (Chen et al. 2011; Wang et al. 2014). Briefly, the patient was a 51‐year‐old Chinese man who presented with paroxysmal weakness in his left limbs 12 days before admission. The weakness progressed to become continuous and was accompanied by speech disturbance. He indulged in excessive eating and drinking before the onset. The patient had no history of hypertension, diabetes mellitus, heart disease, or hyperlipidemia. Macrocephaly was diagnosed during his childhood. Additionally, he was a heavy smoker and drinker for over 20 years. The patient reported having a balanced diet. The family medical history was negative. His neurological examination indicated normal cognition. Gaze palsy, central facial palsy, and hemiparesis on the left side were noticed. Deep tendon reflexes were normal, but bilateral Chaddock signs were positive (Table 1).

**FIGURE 2:**
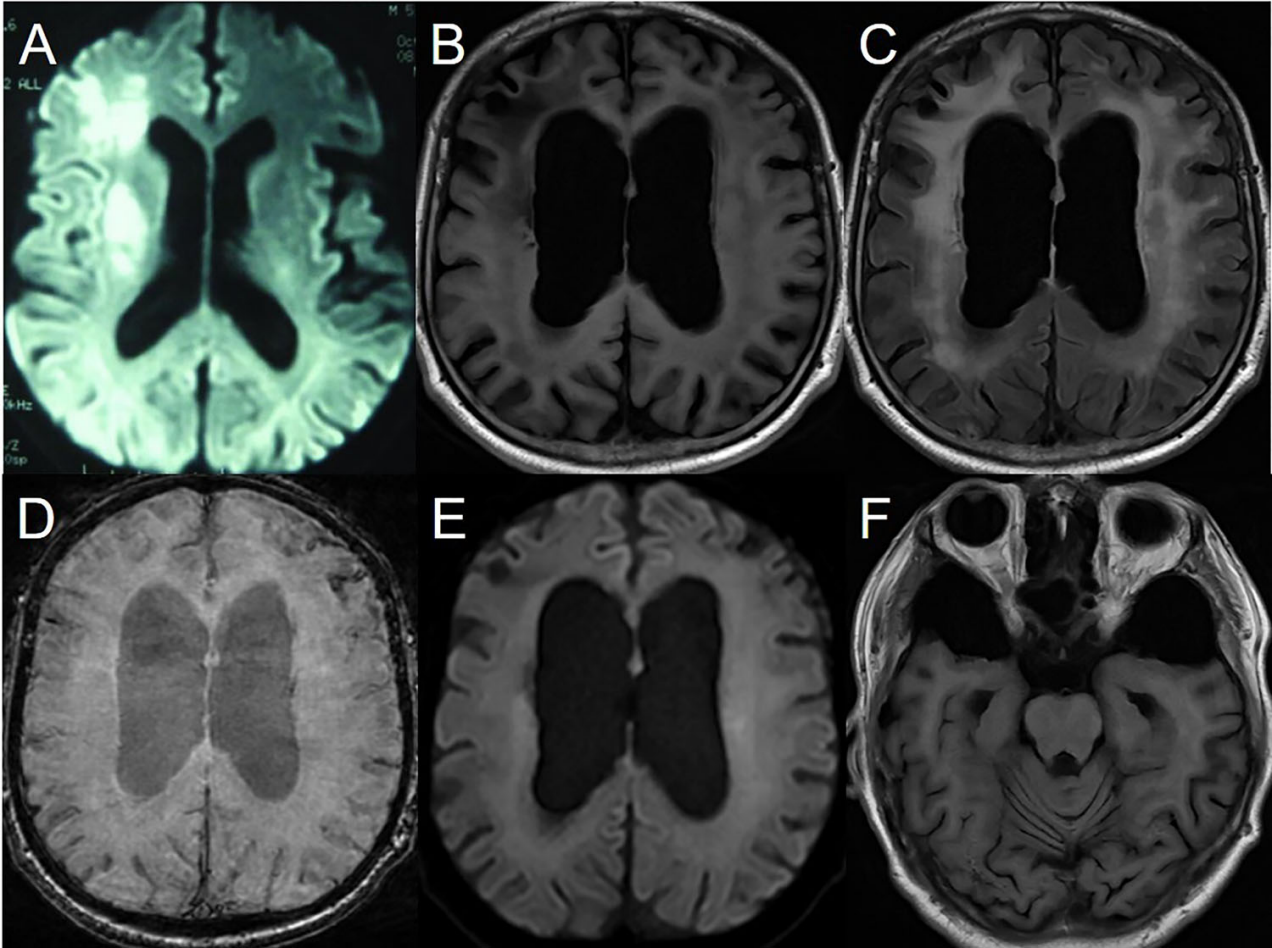
Brain imaging of Patient 2. (A) Restricted diffusion in the right frontal lobe, occipital lobe, and basal ganglion at the age of 51. (B–F) Brain magnetic resonance imaging at age 64 showed multiple subependymal nodules in lateral ventricles and septum pellucidum, predominantly at the roof level. The nodules showed isointensity on susceptibility‐weighted imaging (D) and diffusion‐weighted imaging (E). Periventricular, subcortical, and deep white matter changes were remarkable (C). U‐fibers were involved, but this could be confounded by ischemic lesions. There were multiple infarctions in the right frontal lobe, occipital lobe, and putamen (not shown). Moderate frontotemporal hypoplasia (F), brain atrophy, and ventricular enlargement were noticed.

Routine tests were unremarkable. Low‐density lipoprotein cholesterol was 2.87 mmol/L. Blood total HCY (66 µmol/L) was elevated and vitamin B12 (70 pmol/L) decreased. His *MTHFR* genotype was 677TT. Brain MRI showed restricted diffusion in the right frontal lobe, occipital lobe, and basal ganglion (Figure 2). Several subependymal nodules were noticed in the anterior ventricular horn and on the septum pellucidum without enhancement. Moderate frontotemporal hypoplasia and brain atrophy were present (Table 2). Cervical vascular ultrasound and head magnetic resonance angiography found a narrow left intracranial vertebral artery throughout its course. Holter monitor and echocardiogram detected no heart arrhythmia or structural lesions. An elevation of urine GA and 3‐OH‐GA was found. Subsequent gene test detected heterozygous variants of c.533G> A, p.(Gly178Glu) and c.1205G> A, p.(Arg402Gln) in the *GCDH* gene, which were inherited from his mother and father, respectively. No novel variations were found (Table 1). No other variations consistent with the phenotype were found.

He was treated with a low‐lysine diet and l‐carnitine. Folic acid, vitamin B6, and mecobalamin were given for HHcy. The patient was followed up after 13 years. Left hemiparesis remained but no stroke‐like episode had recurred since then. However, his cognitive function declined significantly. While creatinine was still normal, the albumin‐to‐creatinine ratio increased to 139.39 mg/g, and 24‐h urine protein 1 g/24 h. Blood ammonia (69 µmol/L) was elevated. Total HCY (7 µmol/L) and vitamin B12 (216 pmol/L) returned to normal after treatment. EEG showed a slow background with bilaterally regional continuous slow activity, and sharp waves in the right central region (Table 1). A follow‐up brain MRI after 13 years revealed the presence of multiple subependymal nodules, which were consistent with the nodules observed in the initial imaging. Additionally, there was a notable presence of leukoencephalopathy, primarily located in the periventricular areas and deep white matter. Multiple infarctions were observed in the right cerebral hemisphere (Table 2, Figure 2). The magnetic resonance angiography further revealed the occlusion of the left intracranial vertebral artery.

### 3.2 Literature Review

**FIGURE 3:**
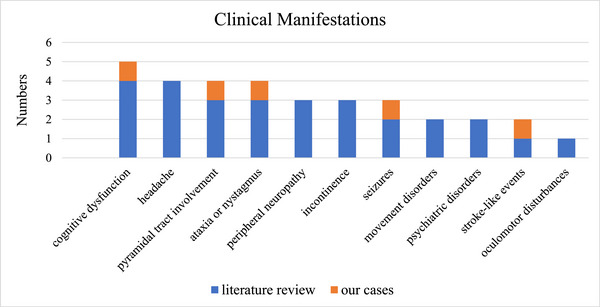
Clinical manifestations of adult‐onset glutaric aciduria Type 1.

A literature review found seven adult‐onset GA‐1 patients (Bähr et al. 2002; Boy, Heringer, Brackmann, et al. 2017; Gelener et al. 2020; Herskovitz et al. 2013; Külkens et al. 2005; Pierson et al. 2015; Sonmez et al. 2007), whose age at onset ranged from 19 to 61 years. The most common initial symptom is headache (4/7). Others include peripheral neuropathy (2/7) and stroke‐like events (1/7). As with other late‐onset GA‐1 patients, adult‐onset type present with nonspecific neurological symptoms or signs, including cognitive dysfunction (4/7), headache (4/7), pyramidal tract involvement (3/7), ataxia or nystagmus (3/7), peripheral neuropathy (3/7), incontinence (3/7), movement disorders (2/7), seizures (2/7), psychiatric disorders (2/7), stroke‐like events (1/7), oculomotor disturbances (1/7) (Figure 3). Only one case in the previous literature reported the routine diet (Mediterranean) (Gelener et al. 2020). Besides our patients, only one case mentioned HHcy (Boy, Heringer, Brackmann, et al. 2017) and none showed the result of ammonia. EEGs were mostly nonspecific, with two showing interictal regional epileptiform discharges. Polyneuropathy was detected by nerve conduction studies in 2/4 of the patients. All of the patients were classified as the HE type (Table 1).

To date, subependymal lesions have been reported in only 10 late‐onset cases (Bähr et al. 2002; Boy, Heringer, Brackmann, et al. 2017; Gelener et al. 2020; Harting et al. 2009; Herskovitz et al. 2013; Korman et al. 2007; Külkens et al. 2005; Pierson et al. 2015; Sonmez et al. 2007) (including 5 adult‐onset patients) and 10 early‐onset cases (Boy, Heringer, Brackmann, et al. 2017; Karimzadeh et al. 2015; Patel et al. 2018). The earliest age presenting with subependymal involvement was about 5 months old (Karimzadeh et al. 2015). The lesions are typically solitary or multiple, varying in size from 1 to 19 mm in diameter. Smaller lesions appear as round or semicircular nodules with smooth surfaces, showing both T1 and T2 isointensity, or T1 hypointensity and T2 hyperintensity compared to normal white matter. Larger lesions may have a “cauliflower” appearance, irregular in shape, and may be contiguous between lesions. Signals are often heterogeneous with punctate hypointensity on susceptibility‐weighted imaging, and there may be nodular or linear enhancement on the surface. Some lesions contain cysts with hyperintense T2, hypointense T2‐fluid attenuated inversion recovery, and hyperintense apparent diffusion coefficient changes. Subependymal lesions are usually located at the top of the lateral ventricles, with more on the medial wall and septum pellucidum, resembling “stalactites.” As the disease progresses, the lesions gradually increase or enlarge in three patients. The size of subependymal lesions seems to be associated with age at onset and age at imaging, but not with sex or urine GA (Table 2).

White matter lesions in adult‐onset GA‐1 are typically significant, patchy, and primarily located in periventricular areas and deep white matters with a Fazekas grade of 2–3. Since most cases did not report the U‐fibers, we could only interpret from the given images and found that 5/7 spared the U‐fibers. Gray matter abnormalities account for 1/5 of nonstroke cases. All patients showed mild‐to‐severe frontotemporal hypoplasia (5/5) and mild‐to‐moderate brain atrophy (5/5). Additionally, 1/5 of the patients showed hydrocephalus on brain MRI (Table 2).

**FIGURE 4:**
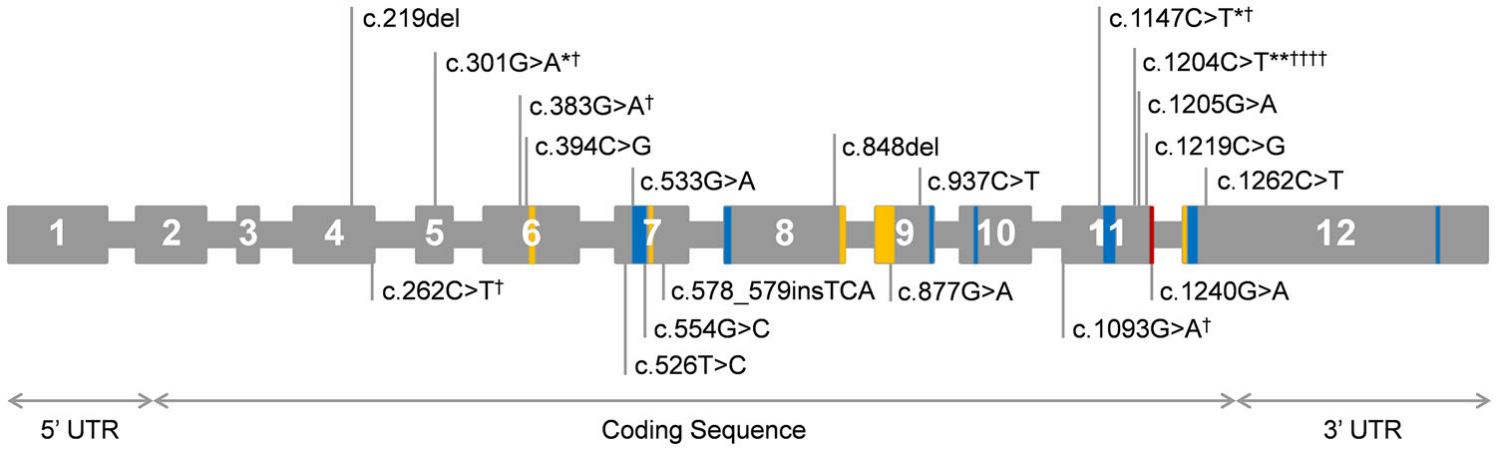
*GCDH* variants of adult‐onset glutaric aciduria Type 1 and patients with subependymal lesions. (1) Variants on the top half are those detected in adult‐onset glutaric aciduria type 1 (GA‐1). * stands for one repeated variant. (2) All variants shown are linked to GA‐1 patients with subependymal lesions. † stands for one repeated variant. The adult‐onset patient with c.219del and c.394C> G did not have subependymal lesions, but the two variants were associated with another late‐onset patient with subependymal lesions. Color scheme: red, active site; blue, binding site of flavin adenine dinucleotide; orange, binding site of substrate. UTR, untranslated region.

**FIGURE 5:**
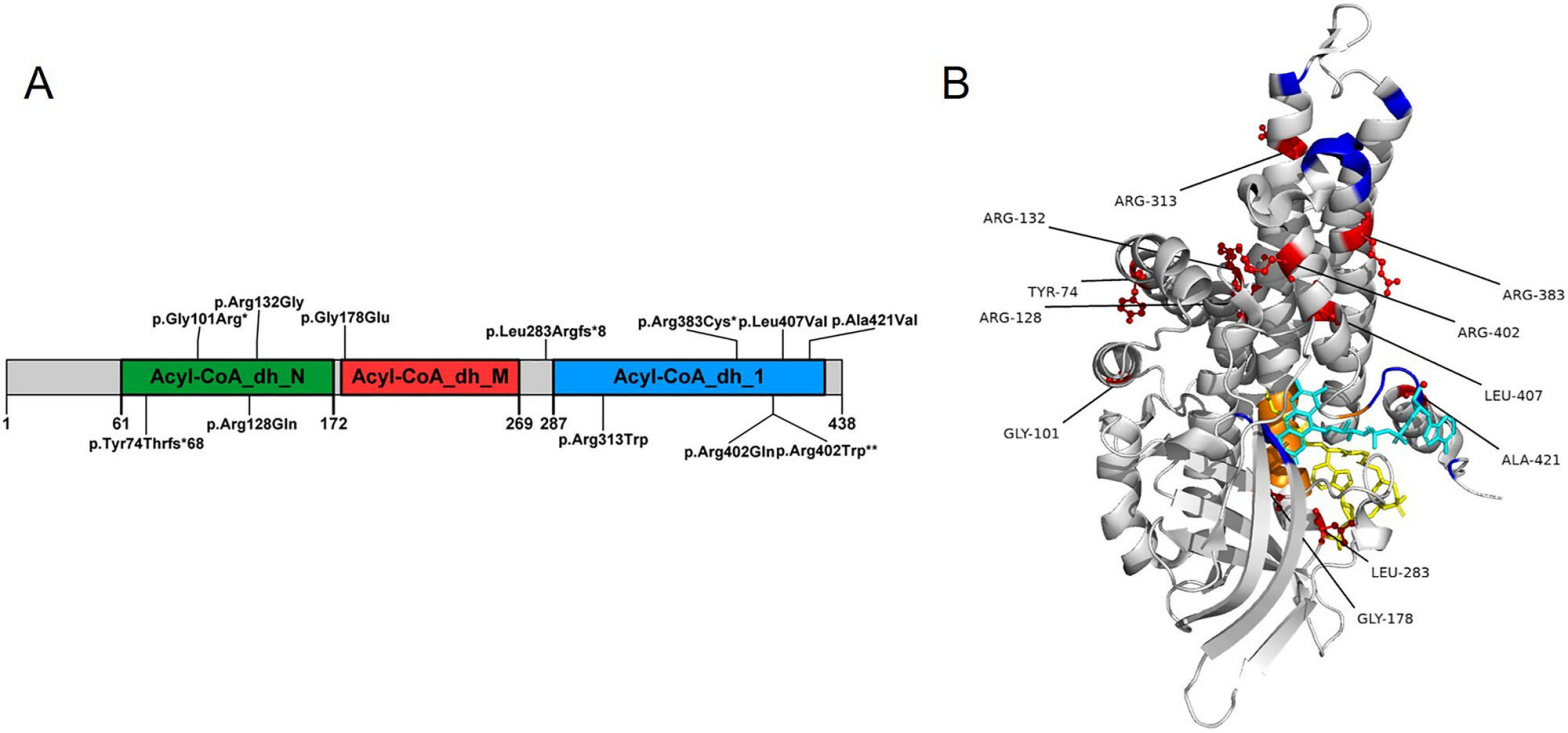
Structural analysis of the GCDH protein (PDB ID: 1SIR). (A) 2D structure of GCDH protein and pathogenic variants of adult‐onset glutaric aciduria Type 1 (GA‐1). (B) 3D structure of GCDH protein and pathogenic variants of adult‐onset GA‐1. Color scheme: red, pathogenic variants of adult‐onset GA‐1; yellow sticks, a flavin adenine dinucleotide (FAD) molecule; cyan sticks, the alternative substrate 4‐nitrobutyryl‐CoA (NBC); blue, binding site of FAD; orange, binding site of NBC.

In adult‐onset GA‐1 patients, a total of 12 variants were identified, including three homozygous and five compound heterozygous variants. The variant sites are primarily located in exon 11. The most common variant is c.1204C> T, p.(Arg402Trp). No specific hotspot variants for adult‐onset GA‐1 were observed. The most common variant remained c.1204C> T, p.(Arg402Trp) (Figure 4). The distribution of the variants included exons 4–12, with exon 11 being the most common. Gene and protein structure illustrations showed that these variants are not located in the flavin adenine dinucleotide (FAD) or 4‐nitrobutyryl‐CoA (NBC) binding sites (Figure 5).

## 4 Discussion

Both of our patients developed symptoms during adulthood, with epileptic seizure and ischemic stroke as the main manifestations, respectively. Brain MRIs revealed subependymal nodules, leukoencephalopathy, and frontotemporal hypoplasia. Elevation of urine 3‐OH‐GA and GA together with high blood C5DC strongly suggested the diagnosis of GA‐1. Subsequent gene tests further confirmed the diagnosis (Boy et al. 2023).

We report for the first time a case of adult‐onset GA‐1 presenting with seizures at onset. The patient had generalized motor seizures with no focal semiological features or EEG changes and a negative family history. Since a detailed neurological work‐up did not reveal any other explainable causes, a possible relationship between GA‐1 and the seizures was suspected. Seizures are not uncommon in GA‐1. A study of 101 Chinese patients showed that up to 40% (40/101) had seizures (E et al. 2021). Another international multicenter study found that in late‐onset patients without acute encephalopathic crisis, 19% (5/26) presented with seizures at onset (Kölker et al. 2015). Including our two new cases, 33% (3/9) of adult‐onset patients experienced seizures. The mechanism of seizure in GA‐1 remains unclear. Abnormal accumulation of organic acids may cause increased release of glutamate by inhibiting glutamate decarboxylase, and may also reduce GABA release, leading to cortical hyperexcitability (Pasquetti et al. 2017). However, this epileptogenicity might be transient, as Patient 1 remained seizure free for 2 years after withdrawal of antiseizure medications. It is noteworthy that valproic acid may exacerbate seizures in GA‐1. One possible explanation is secondary carnitine deficiency due to valproic acid, which affects glutaric acid metabolism (McClelland et al. 2009). Therefore, GA‐1 should also be considered besides mitochondrial diseases as a possible cause of genetic metabolic disorders aggravated by valproic acid.

The second patient in this article presented with recurrent stroke‐like events. Although the patient had a history of smoking, alcohol drinking, and HHcy, there was no clear evidence of large artery atherosclerosis based on vascular imaging. No cardiac or systemic diseases were found, hence no evidence of cardioembolic or other common causes. Considering the patient's history of excessive eating and alcohol consumption before the onset, and the nonarterial distributed restricted diffusion, metabolic stroke was suspected. Metabolic stroke or stroke‐like episodes refer to acute focal brain injury due to metabolic disorders in the absence of large vessel rupture or obstruction (Mastrangelo et al. 2022). Different from ischemic stroke, it is mediated by the accumulation of toxic metabolites leading to mitochondrial dysfunction. This will further trigger neuronal edema, leading to circulatory dysfunction and cerebral tissue ischemia (Zinnanti et al. 2014). Several organic acidopathies can lead to metabolic stroke, such as methylmalonic academia, propionic academia, isovaleric academia, and GA‐1 (Mastrangelo et al. 2022). Only one previous report has been found describing an adult‐onset patient with stroke‐like episodes (Boy, Heringer, Brackmann, et al. 2017). Studies have shown that during the chronic phase of GA‐1, patients have a slow middle cerebral artery blood flow velocity, an increased mean transit time, and an increased cerebral blood flow, indicating dilation of small arteries and an increase of perfusion to meet metabolic needs. However, this critical state is susceptible to a supply–demand imbalance of energy and might end up with metabolic stroke following certain triggers, such as excessive eating and alcohol consumption as in our case (Strauss, Donnelly, and Wintermark 2010). The identification of this type of stroke is crucial because its key treatment is not antithrombosis but rather increasing energy supply, reducing energy demand, and increasing cerebral perfusion pressure (Strauss, Donnelly, and Wintermark 2010).

An interesting finding is that both of our patients were accompanied by remarkable HHcy and mild hyperammonemia. HHcy itself does not cause seizures. HHcy leads to stroke by promoting atherosclerosis, which was not considered as the etiology of stroke in Patient 2. Therefore, HHcy cannot explain the clinical manifestations of the two cases. Investigation of the *MTHFR* gene revealed a TT genotype. No other causes for HHcy (Al Mutairi 2020; Moll and Varga 2015) were identified. However, reduction in MTHFR enzyme activity alone is usually insufficient to cause severe elevation of HCY (de Bree et al. 2003; Moll and Varga 2015). The serum vitamin B12 levels of both patients were significantly lower than normal, suggesting an important role of dietary factors in the development of HHcy. The relationship between GA‐1 and HHcy is unclear. Since the metabolic pathways of GA and HCY have no intersection, their combination might be a coincidence. However, we notice that there is a significant overlap between foods rich in vitamin B12, lysine, and tryptophan. Our patients were likely to have a low lysine and tryptophan diet at the same time, which is protective for GA‐1 leading to the delayed onset of the disease. Interestingly, a previously published literature reported a case with HHcy similar to our Patient 2. The patient, starting at the age of 62, exhibited recurrent stroke‐like episodes that did not conform to the typical arterial vascular distribution. No other ischemic stroke risk factors were found apart from HHcy (Boy, Heringer, Brackmann, et al. 2017). We speculate that HHcy might contribute to the metabolic disturbance of GA‐1 resulting in certain phenotypes. Based on studies in rat GA‐1 models, glutarylation of arginase‐1 may inhibit urea cycling under high lysine intake, leading to hyperammonemia (Gonzalez Melo et al. 2021). Some authors have reported elevated blood ammonia in early‐onset GA1 (Banikazemi, Mazidi, and Nematy 2014; Sadehal and Eshraghi 2019). Our patients had no evidence of common hyperammonemia etiologies. It seems reasonable that adult‐onset GA‐1 patients could also have urea cycle defects. This finding may be significant for understanding the pathophysiological mechanisms of the disease. Further research is warranted to establish the role of HCY and urea cycle in GA‐1.

The subependymal lesion is a characteristic radiological finding in late‐onset GA‐1 (Boy et al. 2023). Its prevalence in adult‐onset patients is approximately 78% (7/9) including our two new cases. To date, no pathological data on subependymal lesions have been found. According to the pathogenesis and radiological findings, it is suggested that the accumulation of metabolites such as GA and 3‐OH‐GA in the brain might be responsible for this phenomenon (Tuncel et al. 2018). We found that patients with later onset or older age at the time of radiological examination appeared to have larger lesions. Additionally, even with early diagnosis and treatment, subependymal lesions could still be detected by MRI in adults. These findings suggest that subependymal involvement may reflect the natural progression of the disease (Patel et al. 2018). Leukoencephalopathy primarily locates in periventricular areas and deep white matter. As the disease progresses, white matter lesions can slowly worsen. Gray matter lesions are less common than early‐onset cases. Frontotemporal hypoplasia and brain atrophy were present in all patients with varying severity. These suggest that adult‐onset GA‐1 shares some common imaging features with non–adult‐onset patients, but also possesses unique characteristics that should be given clinical attention.

The correlation between GA‐1 genotype, biochemical phenotype, and clinical phenotype is complex. We identified 12 genetic variants associated with adult‐onset GA‐1. The c.1219C> G variant was only reported in one case of an adult‐onset patient (Pierson et al. 2015), while the other variants were observed in patients with late‐onset (Anikster et al. 1996; Harting et al. 2009; Fraidakis et al. 2015; Gupta et al. 2015; Boy, Heringer, Brackmann, et al. 2017) or early‐onset forms (Anikster et al. 1996; Hou et al. 2007; Strauss et al. 2007; Wang et al. 2014; Gupta et al. 2015; Kurkina et al. 2020), some of which could present as acute encephalopathy. However, there is no clear correlation between these variants and specific clinical phenotypes, nor can they predict early or late onset. Previous studies have shown that the GA‐1 genotype is to some extent associated with GA excretion and residual enzyme activity (Christensen et al. 2004). HEs have been associated with a higher risk for extrastriatal abnormalities (Boy, Heringer, Brackmann, et al. 2017), subdural hemorrhage (Boy et al. 2021), and worse cognition (Märtner et al. 2021). However, HEs do not necessarily show severe clinical presentations (Boy et al. 2023; Schuurmans et al. 2023). In our study, all variants in adult‐onset GA‐1 were related to HEs except for c.1219C> G (unclassified), c.301G> A (unclassified), and c.1147C> T (both excretors) (Schuurmans et al. 2023). Based on biochemical results, all patients were HEs but presented milder symptoms compared with early‐onset cases. It suggests environmental factors such as diet might be important in shaping the phenotype of each patient, but its influence has not been fully studied yet. Most of the patients with subependymal damage were HEs. One study with (1)H magnetic resonance spectroscopy found that HEs showed significantly increased GA in the white matter compared with LEs (Harting et al. 2015). This suggests HEs might have more accumulation of metabolites in the brain. However, given that many other HEs did not show subependymal lesions, the link between biochemical phenotype and subependymal lesions is still uncertain.

An analysis of 421 pathogenic variants in the *GCDH* gene among 532 patients (including 3 adult‐onset cases) revealed that the variant sites were predominantly located in exons 11 and 12, with relatively high frequencies also observed in exons 6, 8, and 9 (Schuurmans et al. 2023). Our study similarly found that variants in adult‐onset GA‐1 were more frequently located in exon 11. However, for subependymal lesions, exon 7 also exhibited a high frequency. Pathogenic variants tend to cluster around important structural domains of GCDH protein, such as the FAD and substrate binding domain, with amino acid changes in their vicinity correlating with HE and symptomatic clinical phenotypes (Schuurmans et al. 2023). Previous studies have discovered a shared characteristic among variants with residual activities exceeding 10% that they were not located at the binding sites of FAD or the alternative substrate NBC (Yuan et al. 2023). This distribution is similar to that observed in adult‐onset GA‐1. However, other factors such as protein stability may also play an important role in GA‐1. For example, previous studies have shown that p.Met263Val and p.Arg402Trp significantly affect the formation of the GCDH homotetramer (Keyser et al. 2008). In vitro and in silico studies support that higher protein stability may be associated with higher enzymatic activity. However, this study did not find a relationship between protein stability and clinical phenotype (Yuan et al. 2023). Therefore, further functional validation is needed to clarify the impact of variants associated with adult‐onset GA‐1 on protein function.

This study has some limitations. First, the sample size is small. Some of the nonspecific symptoms might be mistaken as the manifestation of GA‐1 due to sampling bias. Since only three patients reported HCY and two reported blood ammonia, we could only provide descriptions instead of conducting statistical analysis. As a result, the phenotypic spectrum of adult GA‐1 in our study is not conclusive. The analysis of genotype–phenotype correlation is similarly limited, with compromised reliability and validity. Further larger‐scale and age‐stratified studies are required to confirm our results. Second, the data quality is inconsistent. Many previous studies failed to report the age of onset and lacked numerical values of metabolic products, or were missing quantitative descriptions of subependymal damage, thereby limiting statistical inferences. Additionally, some cases lacked detailed descriptions of clinical symptoms, potentially affecting the comprehensive summary of the clinical phenotype. Moreover, most reports, including our two cases, did not assess enzyme activity. We suggest that future case reports provide more detailed and consistent information.

## 5 Conclusion

Adult‐onset GA‐1 may present a clinical spectrum. Of particular note, adult‐onset patients may have concomitant HHcy. We speculate that dietary factors might contribute to the late onset of GA‐1 and HHcy, suggesting the role of environmental factors in shaping the phenotype of GA‐1. The subependymal lesion is a characteristic radiological finding in adult‐onset cases, which should trigger an investigation for GA‐1. All adult‐onset GA‐1 patients are HE, but no specific hotspot variants have been found. All *GCDH* variants are located in nonactive binding regions. Our study provides useful insights for clinical practice in adult neurology and for future research on how environmental and genetic factors influence the clinical phenotype of GA‐1.

## Author Contributions

**Luhua Wei**: validation, formal analysis, supervision, writing–review and editing, resources, conceptualization, methodology, writing–original draft. **Jieyu Li**: investigation, formal analysis, visualization, project administration, resources, writing–original draft, software. **Zhiying Xie**: methodology, software, data curation. **Ying Zhu**: methodology, software, data curation. **Jing Chen**: resources, data curation, investigation. **Yawen Zhao**: investigation, resources, data curation. **Yun Yuan**: writing–review and editing, supervision. **Yining Huang**: writing–review and editing, supervision. **Yanling Yang**: supervision, writing–review and editing. **Zhaoxia Wang**: conceptualization, methodology, supervision, writing–review and editing. **Jing Chen**: conceptualization, writing–review and editing, methodology, supervision, resources.

## Ethics Statement

This study has been approved by the health authority ethical committee of Peking University First Hospital. The study was performed in accordance with the ethical standards as laid down in the 1964 Declaration of Helsinki and its later amendments or comparable ethical standards.

## Consent

Informed consent was obtained from all individual participants included in the study. The authors affirm that human research participants provided informed consent for the publication of their data.

## Conflicts of Interest

The authors declare no conflicts of interest.

### Peer Review

The peer review history for this article is available at https://publons.com/publon/10.1002/brb3.70281.

## Data Availability

The data that support the findings of this study are available on request from the corresponding author. The data are not publicly available due to privacy or ethical restrictions.
